# The LEF1–LAG3 axis regulates CD4^+^ T cell function during *Plasmodium yoelii* NSM infection

**DOI:** 10.1186/s13071-026-07439-5

**Published:** 2026-05-26

**Authors:** Wenbo Peng, Guikuan Liang, Keyu Lu, Feng Mo, Xiongyu Xie, Mingjie Chen, Haiwen Yuan, Lixin Luo, Xingyue Wang, Long Xu, Haixia Wei, Lu Li, Shan Zhao, Hongyan Xie, Xingfei Pan, Jun Huang

**Affiliations:** 1 Department of Infectious Diseases, Key Laboratory for Major Obstetric Diseases of Guangdong Province, The Third Affiliated Hospital, Guangzhou Medical University, Guangzhou, China; 2 Key Laboratory of Immunology, Sino-French Hoffmann Institute, School of Basic Medical Sciences, Guangzhou Medical University, Guangzhou, China; 3 Department of Laboratory Medicine, The Sixth Affiliated Hospital of Guangzhou Medical University, Qingyuan People’s Hospital, Qingyuan, China; 4 Guangdong Provincial Key Laboratory of Allergy and Clinical Immunology, The Second Affiliated Hospital, Guangdong Provincial Key Laboratory of Allergy and Clinical Immunology, Guangzhou Medical University, Guangzhou, China; 5 The Department of Critical Care Medicine, General Hospital of Southern Theater Command of PLA, Guangzhou, 510010 Guangdong China

**Keywords:** Malaria, CD4⁺ T cells, LAG3, LEF1

## Abstract

**Background:**

CD4⁺ T cells are pivotal in coordinating anti-malarial immunity, while co-inhibitory receptors such as LAG3 critically regulate their function. However, the phenotype of LAG3⁺CD4⁺ T cells during *Plasmodium* infection and the upstream molecular mechanisms regulating LAG3 expression remain incompletely elucidated.

**Methods:**

We established a murine model using *Plasmodium yoelii* NSM (*P. yoelii* NSM). A multifaceted approach, incorporating single-cell RNA sequencing (scRNA-seq), flow cytometry, magnetic bead-based cell sorting, real-time quantitative polymerase chain reaction (RT–qPCR), dual-luciferase reporter assays, and in vitro cultures with the Wnt agonist CHIR99021, was employed. We characterized splenic CD4⁺ T cell dynamics, the phenotypic and functional profiles of LAG3⁺CD4⁺ T cells, and the transcriptional regulatory relationship between lymphoid enhancer-binding factor 1 (LEF1) and *Lag3*.

**Results:**

*Plasmodium yoelii* NSM infection induced significant splenomegaly and remodeling of the splenic CD4⁺ T cell compartment, with increased absolute numbers of CD4⁺ T cells, upregulated activation markers (ICOS, CD69), downregulated naïve marker CD62L, and enhanced secretion of IL-10 and IFN-γ. Both scRNA-seq and flow cytometry confirmed that infection markedly upregulated LAG3 on CD4⁺ T cells. These LAG3⁺CD4⁺ T cells exhibited an activated phenotype, characterized by increased proliferative capacity (Ki67⁺), an increased proportion of the effector phenotype (CD44ʰⁱCD62Lˡᵒ), and concurrent upregulation of multiple co-inhibitory receptors (PD-1, TIM-3, TIGIT). Mechanistically, LEF1 expression was significantly downregulated in CD4⁺ T cells post infection. Dual-luciferase reporter assay demonstrated that LEF1 directly binds to the *Lag3* promoter, acting as a transcriptional repressor. Furthermore, treatment with the Wnt agonist CHIR99021, which stabilizes the upstream signaling of LEF1, dose-dependently reduced the frequency of LAG3⁺CD4⁺ T cells.

**Conclusions:**

This study suggests that the LEF1–LAG3 axis is involved in modulating CD4⁺ T cells during *P. yoelii* NSM infection. LAG3⁺CD4⁺ T cells exhibit an activated phenotype with regulatory potential, which may contribute to balancing anti-parasitic immunity and immunopathology. These findings suggest that modulating LEF1-mediated transcriptional repression of *Lag3* offers a promising avenue for fine-tuning anti-malarial immune responses.

**Graphical Abstract:**

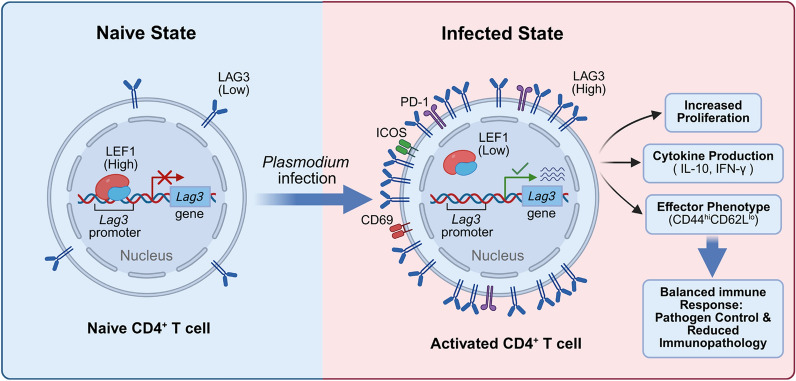

**Supplementary Information:**

The online version contains supplementary material available at 10.1186/s13071-026-07439-5.

## Background

Malaria persists as a leading global cause of infectious disease mortality. According to World Health Organization (WHO) 2025 data, this disease caused about 282 million clinical cases and 610,000 deaths across 80 endemic countries [1]. *Plasmodium* infection in humans is strictly demarcated into two distinct phases: the clinically silent liver stage and the symptomatic erythrocytic stage [2]. The liver stage begins with sporozoite inoculation into the skin and culminates in merozoite release into the bloodstream, representing an obligate, clinically silent period of parasite amplification [3, 4]. Transition to the erythrocytic stage occurs upon merozoite release from the liver, which drives all malaria-related clinical manifestations. Unlike the singular liver stage (excluding relapses), the erythrocytic stage perpetuates via continuous, synchronous cycles of erythrocyte invasion, replication, and rupture, persisting until host death or parasite clearance [5, 6]. All malaria-related clinical manifestations, including periodic febrile paroxysms, splenomegaly, and hemolytic anemia, originate exclusively from the erythrocytic stage [7]. With the emergence of artemisinin resistance in *Plasmodium* strains, elucidating host immune responses has become imperative for developing next-generation control strategies [8].

CD4⁺ T cells play a pivotal role in malaria immunobiology [9]. During the liver stage, CD4⁺ T cells synergize with IFN-γ to enhance antigen presentation and nitric oxide-mediated parasite clearance [10]. In the erythrocytic stage, activation by dendritic cells and parasite-derived stimuli (e.g., GPI anchors, hemozoin) drives CD4⁺ T cell differentiation into diverse effector subsets [10]. Th1 cells (T-bet⁺IFN-γ⁺) orchestrate acute infection control via macrophage activation, antibody-dependent cellular cytotoxicity (ADCC), and natural killer (NK) cell recruitment [11]. However, dysregulated IFN-γ production impairs B cell memory formation and exacerbates immunopathology through excessive TLR/MyD88 signaling [12, 13]. Notably, while classical models emphasized Th2 cells in humoral immunity, recent evidence suggests that T follicular helper (Tfh) cells, rather than canonical IL-4-producing Th2 cells, are the primary drivers of parasite-specific B cell responses and germinal center formation during malaria [14–16]. In addition, regulatory T (Treg) cells provide a critical break on T cell hyperactivation to maintain self-tolerance [13]. Thus, deciphering CD4⁺ T cell heterogeneity in malaria immunity is vital for rational vaccine design and immunotherapeutic interventions.

The upregulation of co-inhibitory receptors is a hallmark of the host response to *Plasmodium* infection. Both murine and human studies demonstrate sustained expression of PD-1, LAG3, CTLA4, and TIM-3 on T cells [17–20]. Rather than merely signifying exhaustion, this profile serves as a protective feedback mechanism to constrain immunopathology [18, 21]. While immune checkpoint blockade restores T cell function in cancer immunotherapy [22], its role in acute infections like malaria warrants investigation to delineate their therapeutic potential.

LAG3, an inhibitory receptor expressed on activated CD4⁺/CD8⁺ T cells, NK cells, and plasmacytoid dendritic cells (pDCs), is upregulated in effector T cell populations from patients with malaria [19, 23, 24]. In vivo blockade of PD-L1 and LAG3 restored CD4⁺ T cell function; expanded Tfh cells, germinal center B cells, and plasmablasts; enhanced protective antibody responses; and promoted rapid clearance of blood-stage malaria in mice [25–27]. The efficacy of this blockade validates the critical role of co-inhibitory molecules in regulating anti-malarial immunity. Therefore, elucidating the phenotypic properties and regulatory mechanisms of LAG3⁺CD4⁺ T cells post *Plasmodium* infection could yield novel mechanistic insights for developing targeted malaria interventions.

LEF1, a key transcription factor in the Wnt/β-catenin pathway, is essential for T cell development and memory formation [28, 29]. However, how LEF1 directly regulates effector molecules, particularly immune checkpoint receptors such as LAG3, remains poorly understood. This knowledge gap not only represents a fundamental question in T cell immunity against *Plasmodium* infection but also defines the core investigative focus of this study.

In this study, rather than merely characterizing the exhausted phenotype of CD4⁺ T cells, we uncover a previously unappreciated LEF1–LAG3 regulatory mechanism underlying their immunomodulation during *P. yoelii* NSM infection. Mechanistically, we demonstrate that the transcription factor LEF1 governs *Lag3* transcription. The infection-induced suppression of LEF1 directly drives the transcriptional upregulation of LAG3. Ultimately, deciphering this novel LEF1–LAG3 signaling axis provides critical molecular insights and highlights LAG3 blockade as a highly promising therapeutic strategy to potentiate anti-malarial immunity.

## Methods

### Ethical statement

The Institutional Animal Care and Use Committee of Guangzhou Medical University (no. GY2020-134) approved all animal procedures and use and they were conducted in strict accordance with institutional guidelines. Every effort was undertaken to minimize animal suffering throughout the study.

### Reagents and antibodies

The following fluorescently labeled antibodies were purchased from Biolegend (USA): APC anti-mouse CD45 (clone 30-F11, cat. no. 103112), FITC anti-mouse CD3 (clone 145-2C11, cat. no. 100306), PerCP/Cyanine5.5 anti-mouse CD4 (clone GK1.5, cat. no. 100434), FITC anti-mouse CD4 (clone GK1.5, cat. no. 100405), APC anti-mouse LAG3 (clone C9B7W, cat. no. 125210), Brilliant Violet 660™ anti-mouse LAG3 (clone C9B7W, cat. no. 125229), PE/Cyanine7 anti-mouse ICOS (clone C398.4A, cat. no. 313520), APC anti-mouse CD69 (clone H1.2F3, cat. no. 104514), APC anti-mouse IL-10 (clone JES5-16E3, cat. no. 505010), APC anti-mouse IFN-γ (clone XMG1.2, cat. no. 505810), Brilliant Violet 421™ anti-mouse TGF-β (clone TW7-16B4, cat. no. 141408), PE/Cyanine7 anti-mouse PD-1 (clone 29F.1A12, cat. no. 135216), Brilliant Violet 421™ anti-mouse TIM-3 (clone B8.2C12, cat. no. 134011), PE/Cyanine7 anti-mouse TIGIT (clone 1G9, cat. no. 142108), PE/Cyanine7 anti-mouse Ki-67 (clone 16A8, cat. no. 652426), Brilliant Violet 785™ anti-mouse/human CD44 (clone IM7, cat. no. 103041). The Alexa Fluor^®^ 488 Conjugate LEF1 (clone C12A5, cat. no. 8490) was purchased from Cell Signaling USA. The Roswell Park Memorial Institute (RPMI)-1640 (REF C11875500BT 500 mL), fetal bovine serum (FBS) (cat. no. FS301-02), and penicillin and streptomycin (100×, 5000 U/mL) (ref. 15070-063 100 mL) were purchased from Gibco USA. The ionomycin (cat. no. I3909), myristoyl phorbol ethyl ester (PMA) (cat. no. P1585), and Brefeldin A (BFA) (cat. no. B5936) were purchased from Sigma USA. The transcription factor staining kit (cat. no. 00-5523-00) was purchased from Invitrogen USA. The Fixation/Permeabilization Solution Kit (cat. no. 554714) was purchased from BD USA.

### Experimental animals and *Plasmodium* infection

Female C57BL/6 wild-type mice (6–8-weeks old), maintained under specific pathogen-free (SPF) conditions at the Animal Experiment Center of Guangzhou Medical University (Guangzhou, China), were used in this study (no. GY2020-134). The mice were randomly allocated to control or uninfected and infected groups.

*P. yoelii* NSM was provided by the Malaria Research and Reference Reagent Resource Center (MR4). For experimental activation, the cryopreserved *P. yoelii* stored at −80 °C were rapidly thawed in a 37 °C water bath and immediately inoculated into donor mice via intraperitoneal injection (100 μL/mouse). For parasitemia monitoring and strain validation, from days 2–5 post inoculation, thin blood smears of donor mice were prepared daily, Giemsa stained, and microscopically examined to quantify parasitemia (confirming strain viability). At 10–20% parasitemia (optimal viability window), donor mice were euthanized by retro-orbital bleeding under anesthesia. To establish standardized infection model, the infected red blood cells (iRBCs) from terminal blood collection of donor mice were isolated and enumerated, and adjusted to 5 × 10⁶ iRBCs/mL in sterile PBS (vortex-mixed thoroughly). Thereafter, experimental mice received 200 μL intraperitoneal inocula (1 × 10⁶ iRBCs/mouse) to induce synchronized blood-stage infection. Finally, all the experimental mice were sacrificed between days 12 and 16 post infection and relevant experiments were conducted.

### Preparation of spleen lymphocyte suspension

Following sacrifice, spleens from both cohorts (control and experimental) were aseptically harvested. Each spleen was dissociated through a 200-μm nylon mesh in cold D-Hank’s via mechanical trituration. After centrifugation (600*g*, 4 °C, 5 min), pellets underwent erythrocyte lysis (room temperature (RT), 5–7 min) with quenching by D-Hank’s. Washed cells (600*g*, 4 °C, 5 min) were resuspended in complete RPMI-1640 (10% FBS/1% P/S), filtered, and counted [30].

### Flow cytometry analysis

For the detection of cell surface markers, single-cell suspensions of lymphocytes were stained with fluorescently conjugated antibodies against mouse CD45, CD3, CD4, LAG3, ICOS, CD69, CD62L, PD-1, TIM-3, TIGIT, and CD44 for 30 min at 4 °C in the dark. For intracellular cytokine detection, lymphocytes were stimulated with PMA (20 ng/mL) and ionomycin (1 µg/mL) at 37 °C for 5 h and incubated with BFA (10 µg/mL) for the final 4 h to inhibit cytokines secretion. Following surface staining, cells were fixed and permeabilized using a Fixation/Permeabilization Solution Kit for 20 min at 4 °C in the dark. Subsequently, intracellular staining was performed using antibodies against IL-10, TGF-β, and IFN-γ for 30 min at 4 °C in the dark. To evaluate the expression of LEF1 and Ki67, cells were processed with a transcription factor staining kit and incubated with fluorescently labeled anti-LEF1 and anti-Ki67 antibodies for 30 min at 4 °C in the dark. After staining, cells were washed twice and analyzed by flow cytometry. Data analysis was conducted using CytExpert 2.3 software (Beckman Coulter, USA).

### Magnetic bead sorting of CD4^+^ T cells

Splenic lymphocytes were centrifuged and washed with MACS Buffer. For magnetic labeling, cells (10⁷ per sample) were incubated with anti-CD4 MicroBeads (Miltenyi, 1:10 dilution) for 30 min at 4 °C. After washing, the cells were loaded onto a preconditioned LS Column in a magnetic stand. Following three buffer washes (3 mL each), target cells were eluted with 6 mL buffer (3 mL × two flushes) after column removal from the magnet. Finally, the collected cell purity and viability were confirmed by flow cytometry.

### RNA preparation for real-time PCR

To analyze gene expression, cells were snap-frozen in Trizol (Invitrogen, USA) for RNA extraction. Quantitative PCR (qPCR) was then performed using a CFX96 System (Bio-Rad, USA). Details of the primers used are provided:

**Table:** 

Gene	Forward primer	Reverse primer
*β-actin*	5′-CCGTAAAGACCTCTATGCCAA-3′	5′-GGGTGTAAAACGCAGCTCAGTA-3′
*Lag3*	5′-CCACCTCCTGCTGTTTCTCATCC-3′	5′-TCCCTGGCTCACCTGTCTTCTC-3′
*Lef1*	5′-GCCACCGATGAGATGATCCC-3′	5′-TTGATGTCGGCTAAGTCGCC-3′
*Tcf7*	5′-AACTGGCCCGCAAGGAAAG-3′	5′-CTCCGGGTAAGTACCGAATGC-3′

### Single-cell sequencing

Splenocytes from normal (naïve) and infected mice were obtained according to the protocol mentioned above and CD45^+^ immune cells were sorted by flow cytometry (Beckman MoFlo). Their scRNA-seq data are available in the Sequence Read Archive (SRA) under the BioProject IDs PRJNA889506 and PRJNA889507. Sequences were processed and aligned to the GRCm38 genome using Cell Ranger (v3.1.0) with default parameters. All cells were filtered by quality control conditions: 600 < detection genes < 6000; mitochondrial gene count < 10%; log10(GenesPerUMI) > 0.8 (GenesPerUMI = nFeature_RNA/nCount_RNA). DoubletFinder (v2.0.6) was employed to identify and remove potential doublets. Raw counts were normalized using the NormalizeData function in Seurat (v5.3.0).

### Plasmid construction

The *Lag3* promoter-luciferase reporter plasmid (pGL3-Basic-*Lag3*) and the overexpression plasmids encoding *Lef1* (pCDNA3.1-*Lef1*) and *Tcf7* (pCDNA3.1-*Tcf7*), along with their corresponding empty vectors, were constructed by TsingKe Biological Technology (Beijing, China). The fidelity of all constructs was verified via restriction enzyme digestion and DNA sequencing. The pRL-TK normalization plasmid was kindly provided by Prof. Ming-Sheng Cai (Guangzhou Medical University). Details of the primers used are provided:

**Table:** 

Insert fragment	Forward primer	Reverse primer
pGL-*Lag3*-promote	5′-TCCGGTACTGTTGGTAAAGCCACCGAAACGGTCGCGCTTC-3′	5′-CGGCCGCCCCGACTCTAGAAGCTGGGACCCAGAGCAGAGT-3′
pCDNA3.1-vector	5′-TACGGGCCAGATATA CGCGTTGACATTGATTA TTGACTAG-3′	5′-TTCTCTAGTTAGCCAGAGAGCTCTGCTTATATAGACCTCC-3′
pCDNA3.1-*Tcf7*	5′-ATAGGGAGACCCAAGCTGGCTAGCATGTACAAAG-3′	5′-ATCCGAGCTCGGTACCAAGCTTCTAGAGCACTGTCATCGG-3′
pCDNA3.1-*Lef1*	5′-ATAGGGAGACCCAAGCTGGCTAGCATGCCCCAACTTTCCG-3′	5′-ACGGGCCCTCTAGACTCGAGTCAACAAGCTTCCATCTCCAG-3′

### Dual-luciferase reporter assay

At 48 h post transfection of HEK 293 T cells with the target plasmid using Lipofectamine 3000, the culture medium was aspirated and cells were washed twice with PBS. Each well was incubated with 100 μL of 1× Passive Lysis Buffer for 15 min at 25 °C on an orbital shaker. A 20-μL lysate aliquot was transferred to a 96-well plate, and firefly luminescence was quantified immediately after adding 100 μL Luciferase Assay Reagent II. Subsequently, Renilla luminescence was measured following addition of 100 μL Stop & Glo^®^ Reagent. The normalized experimental value (Firefly/Renilla RLU ratio) was subjected to one-way analysis of variance (ANOVA) with Tukey’s post hoc test.

### Cell culture

Under sterile conditions, spleen lymphocytes were harvested and resuspended in cell culture medium. The resulting suspension was aliquoted into 24-well plates (2 × 10^6^ cells/well). All spleen lymphocytes were stimulated with purified anti-mouse CD3 (1 μg/mL, cat. no. 100302) and purified anti-mouse CD28 (1 μg/mL, cat. no. 102102). For Wnt agonism assays, experimental groups received 1 μM, 5 μM, or 10 μM CHIR99021 (SML1046, Sigma, USA), whereas controls were administered an equivalent volume of dimethyl sulfoxide (DMSO). Complete medium was supplemented to achieve a final volume of 1 mL per well, followed by thorough mixing. Plates were subsequently incubated at 37 °C with 5% CO₂ for 48 h.

### Statistical analysis

The experimental data were plotted and statistically analyzed using GraphPad Prism 9.0.0 software. Data following a normal distribution were analyzed using the unpaired *t*-test. For comparisons among multiple groups, one-way analysis of variance (ANOVA) was performed, followed by the least significant difference (LSD) post hoc test. In cases of heterogeneous variance, data with non-normal distribution or unequal variances were analyzed using the Mann–Whitney *U* test. Statistical significance was defined as follows: ^*^P < 0.05, ^**^P < 0.01, ^***^P < 0.001, ^****^P < 0.0001; no significant differences were defined as ns, *P* > 0.05. All data were visualized as mean ± standard error of the mean (SEM).

## Results

### *Plasmodium* infection remodels splenic CD4⁺ T cell responses in mice

**Fig. 1:**
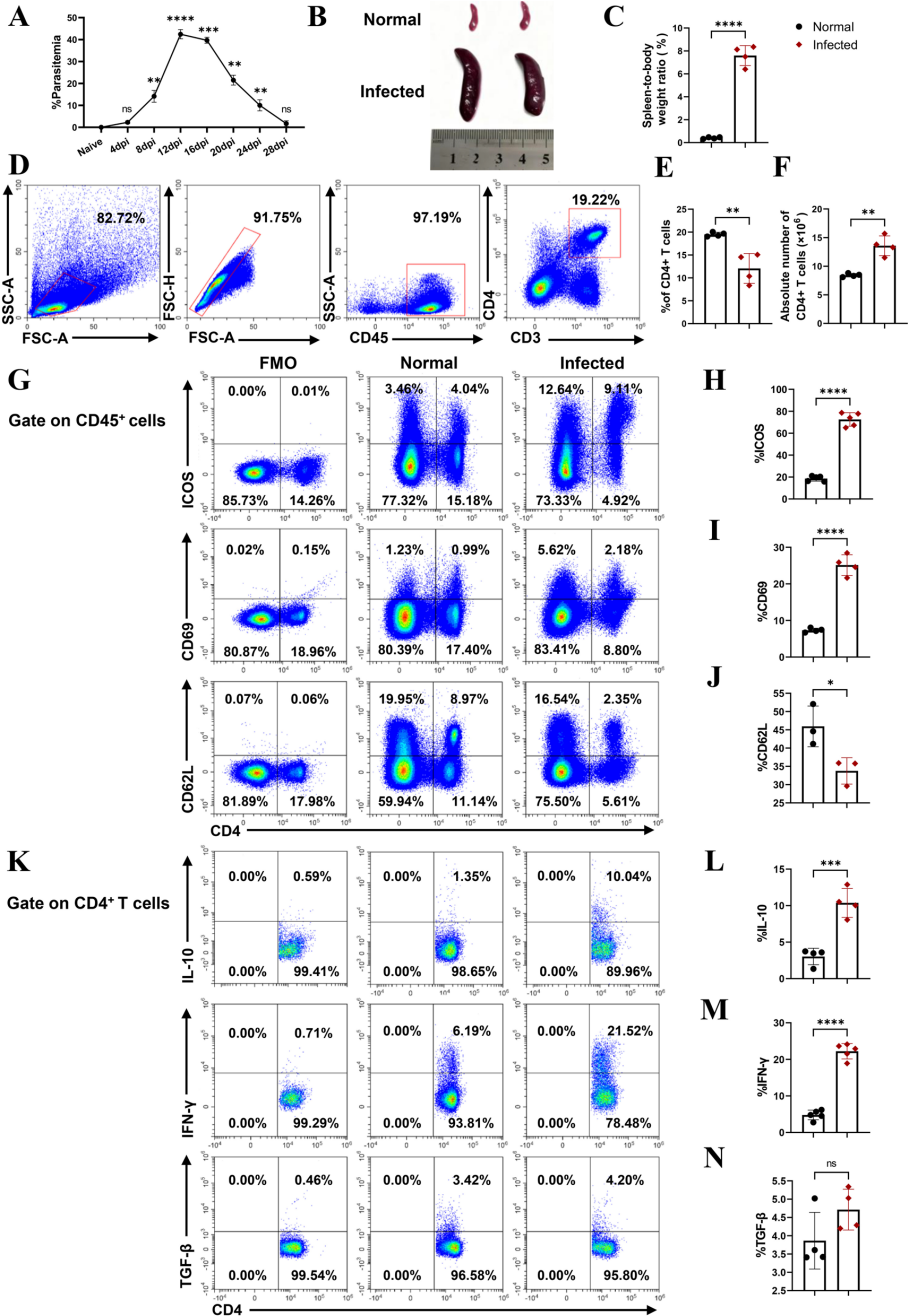
Phenotypic and functional characterization of CD4⁺ T cells in *Plasmodium*-infected mice. **A** Parasitemia monitoring via Giemsa-stained tail vein blood smears every 4 days post infection. **B** Representative splenic morphology of normal and infected mice. **C** Spleen-to-body weight ratio of mice. **D**–**F** The percentage and absolute numbers of splenic CD4⁺ T were measured by flow cytometry. **G**–**J** The expression of ICOS, CD69, and CD62L in splenic CD4⁺ T cells. **K**–**N** The expression of IL-10, IFN-γ, and TGF-β in splenic CD4⁺ T cells. (**A**, **C**, **E**, **F**, **H**–**J**, **L**–**N**) *n* = 3–5 samples per group. Data are shown as the mean ± standard deviation (SD) of three independent experiments. ^*^P < 0.05; ^**^P < 0.01; ^***^P < 0.001; ^****^P < 0.0001; ns, not significant, *P* > 0.05. **A** One-way ANOVA. **C**, **E**, **F**, **H**–**J**, **L**–**N** Two-tailed unpaired *t*-test

To investigate the effect of *P. yoelii* NSM infection on splenic immune responses in mice, we established a corresponding infection model via intraperitoneal injection of *P. yoelii* NSM-iRBCs. Parasitemia was monitored every 4 days by microscopic examination of tail vein blood smears (Fig. 1A). As the parasite load peaked between days 12 and 16 post infection, all subsequent experiments were performed at 12 days post infection (12 dpi). At this time point, spleens from infected mice exhibited significant splenomegaly and darkening, accompanied by a markedly elevated spleen-to-body weight ratio compared with the control group (Fig. 1B, C). These observations are indicative of splenic sequestration of *Plasmodium* parasites and profound pathological alterations in the splenic microenvironment.

Given the central role of CD4⁺ T cells in adaptive immunity [31, 32], we further analyzed the impact of *Plasmodium* infection on this subset. Although the proportion of CD4⁺ T cells among lymphocytes decreased slightly in infected mice (Fig. 1D, E), their absolute numbers increased significantly (Fig. 1F), indicating that infection promotes a numerical expansion of splenic CD4⁺ T cells. To characterize the activation status of these cells, we detected surface expression of activation-associated molecules. Phenotypic analysis revealed that the activation markers ICOS and CD69 were significantly upregulated, while the naïve marker CD62L was markedly downregulated in infected mice (Fig. 1G–J), reflecting a robust activation state of splenic CD4⁺ T cells. Furthermore, analysis of cytokine secretion capacity revealed significantly increased proportions of splenic CD4⁺ T cells producing IL-10 and IFN-γ in the infected group, while TGF-β secretion remained unchanged (Fig. 1K–N). These findings suggest that splenic CD4⁺ T cells undergo extensive phenotypic and functional remodeling during the course of *P. yoelii* NSM infection.

### *Plasmodium* infection induces LAG3 expression on splenic CD4⁺ T cells

**Fig. 2:**
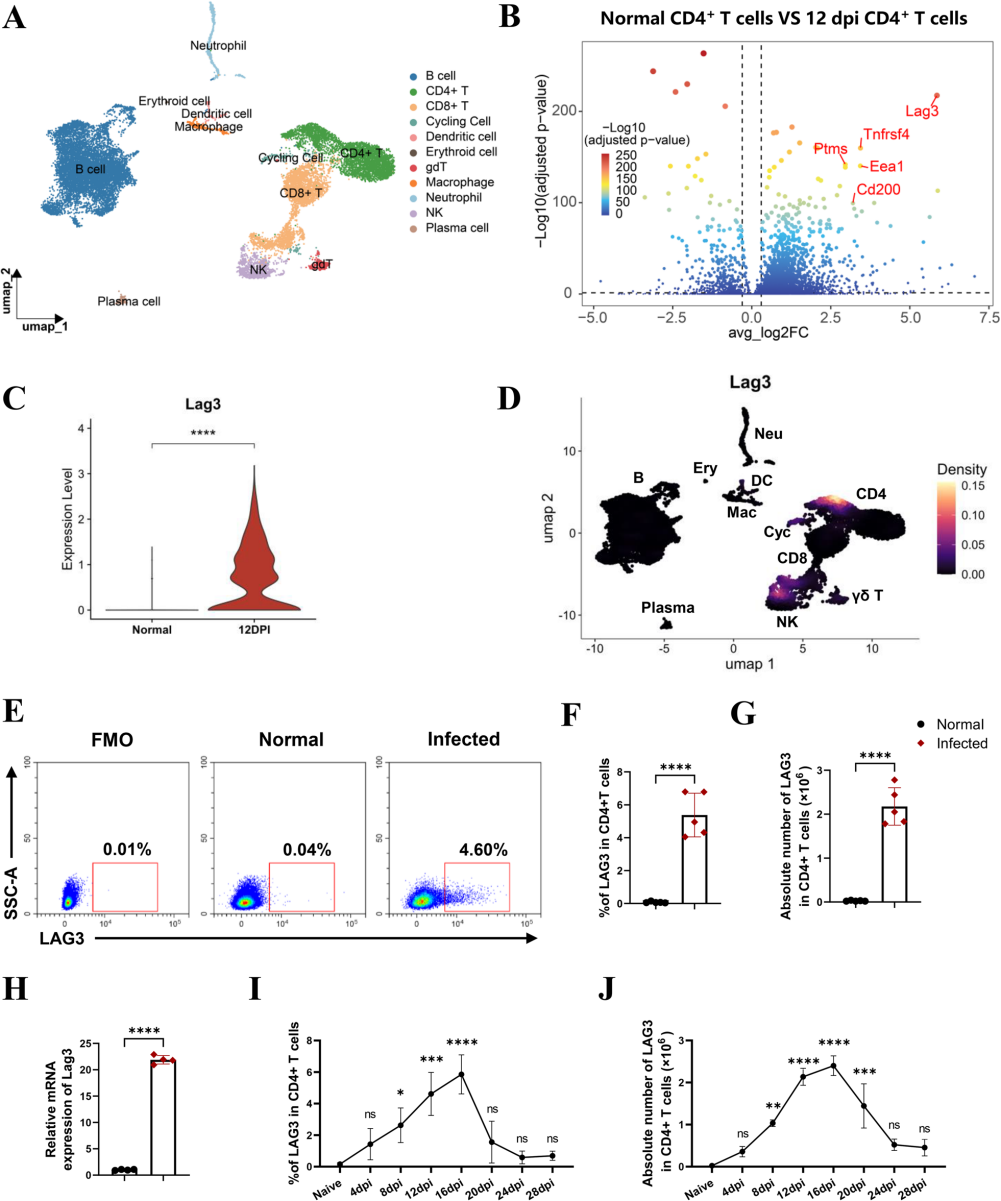
*Plasmodium* infection induces LAG3 expression on CD4⁺ T cells in mice. **A** UMAP projection of splenic lymphocytes from scRNA-seq data. **B** Volcano plot of DEGs in splenic CD4⁺ T cells between the normal and 12 dpi groups. **C** Violin plot of *Lag3* expression in CD4⁺T cells from scRNA-seq data between normal and 12 dpi groups. **D** UMAP visualization showed *Lag3⁺* cells among immune cells. **E**–**G** The percentage and absolute numbers of splenic LAG3⁺CD4⁺ T cells were measured by flow cytometry. **H** *Lag3* mRNA level in splenic CD4⁺ T cells of normal and infected mice. **I**, **J** Temporal changes in LAG3⁺CD4⁺ T cell proportions and counts. **F**–**J** *n* = 3–4 samples per group. Data are shown as the mean ± SD of three independent experiments. ^*^P < 0.05; ^**^P < 0.01; ^***^P < 0.001; ^****^P < 0.0001; ns, no significant, *P* > 0.05. **C** Wilcoxon test; **F**–**H** Two-tailed unpaired *t*-test. **I** and **J** One-way ANOVA

To characterize infection-induced changes at single-cell resolution, scRNA-seq was performed on splenic lymphocytes at 12 dpi. uniform manifold approximation and projection (UMAP) visualization showed that the spleen identified multiple immune cell subsets, including B cells, plasma cells, neutrophils (Neu), erythroid cells (Ery), dendritic cells (DC), macrophages (Mac), cycling cells (Cyc), CD4⁺ T cells, CD8⁺ T cells, NK cells, and γδT cells (Fig. 2A). Given the critical role of CD4⁺ T cells in anti-*Plasmodium* immunity [33, 34], heat-map analysis was used to evaluate transcriptomic alterations in this compartment. At 12 dpi, CD4⁺ T cells exhibited widespread upregulation of cytotoxic genes (e.g., *Gzmb*, *Gzmk*), co-stimulation-associated genes (e.g., *Tnfrsf4*, *Tnfsf8*, *Tnfsf9*), and immune checkpoint genes (e.g., *Lag3*, *Pdcd1*, *Tigit*) (Supplementary Fig. S1A). Volcano-plot analysis confirmed *Lag3*, an inhibitory receptor, was among the most significantly upregulated genes in infected mice (Fig. 2B).

Consistent with these findings, *Lag3* expression was significantly elevated in CD4⁺ T cells at 12 dpi (Fig. 2C), with UMAP visualization illustrating its enrichment within specific activated clusters (Fig. 2D). To validate these transcriptomic results, we employed flow cytometry and RT–qPCR. Both the proportion and absolute number of LAG3⁺CD4⁺ T cells increased significantly in infected spleens (Fig. 2E–G). *Lag3* mRNA levels were similarly upregulated (Fig. 2H). Longitudinal monitoring demonstrated that the expansion of LAG3⁺CD4⁺ T cells followed a time-dependent manner, peaking at 12–16 dpi in alignment with parasitemia kinetics (Fig. 1A; Fig. 2I, J). Therefore, these results demonstrate that *P. yoelii* NSM infection leads to a significant upregulation of LAG3 expression on splenic CD4⁺ T cells.

### Splenic LAG3⁺CD4⁺ T cell remodeling reveals a pivotal immunoregulatory role during *Plasmodium* infection

**Fig. 3:**
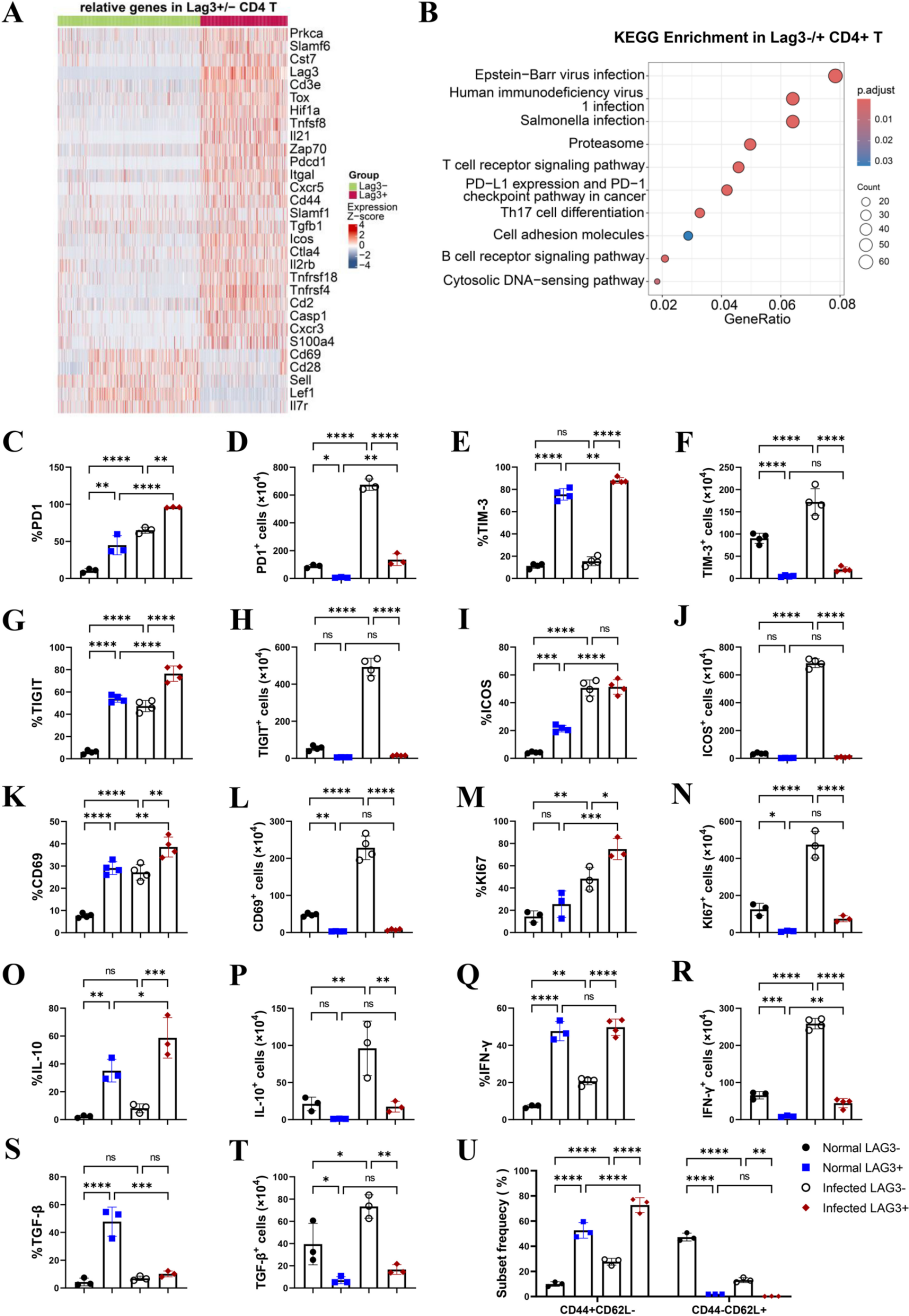
Splenic LAG3⁺CD4⁺ T cell remodeling reveals pivotal immunoregulatory role in *Plasmodium*-infected mice. **A** Heat map of DEGs between LAG3⁻ and LAG3⁺CD4⁺ T cells. Each column represents an individual single cell, and each row represents a gene. **B** KEGG pathway enrichment of DEGs between LAG3⁻ and LAG3⁺CD4⁺ T cells. **C**–**N** The frequencies (%) and absolute cell numbers (×10^4^) are shown for PD-1 (**C, D**), TIM-3 (**E, F**), TIGIT (**G, H**), ICOS (**I, J**), CD69 (**K, L**), and Ki-67 (**M, N**) in LAG3⁻ and LAG3⁺ CD4⁺ T cells. **O**–**T** The frequencies (%) and absolute cell numbers (×10^4^) of IL-10 (**O, P**), IFN-γ (**Q, R**), and TGF-β (**S, T**) in LAG3⁻ and LAG3⁺ CD4⁺ T cells. **U** The percentage of effector (CD44^hi^CD62L^lo^) and resting phenotypes (CD44^lo^CD62L^hi^) in LAG3⁻ and LAG3⁺ CD4⁺ T cells. **C**–**U** *n* = 3–4 samples per group. Data are shown as the mean ± SD of three independent experiments. ^*^P < 0.05; ^**^P < 0.01; ^***^P < 0.001; ^****^P < 0.0001; ns, no significant, *P* > 0.05. **C**–**U** One-way ANOVA

To systematically analyze the phenotypic and functional characteristics of LAG3⁺CD4⁺ T cells during *Plasmodium* infection, the following analyses were conducted. Differential gene expression (DGE) analysis between LAG3⁻CD4⁺ and LAG3⁺CD4⁺ T cells revealed extensive transcriptomic reprogramming (Fig. 3A). Among them, T cell activation and co-stimulation-related genes (e.g., *Icos*), chemokine receptors (e.g., *Cxcr3*, *Cxcr5*), and other immune checkpoints (e.g., *Pdcd1*, *Ctla4*) were significantly upregulated in LAG3^+^ groups. In contrast, naïve makers (e.g., *Il7r*, *Lef1*) and the co-stimulatory molecule *Cd28* were downregulated in the LAG3⁺ group (Fig. 3A). Kyoto Encyclopedia of Genes and Genomes (KEGG) and Gene Ontology (GO) pathway enrichment analyses confirmed that these DEGs were mainly enriched in immune-related processes, including “T cell receptor signaling pathway” and “regulation of T cell activation” (Fig. 3B; Supplementary Fig. S1B). These results showed that LAG3⁺CD4⁺ T cells are involved in multiple key immune responses.

Furthermore, compared with LAG3^−^ and naïve controls, the frequency of PD-1, TIM-3, and TIGIT was significantly elevated in infected LAG3⁺CD4⁺ T cells (Fig. 3C, E, G; Supplementary Fig. S2A). While the *Icos* mRNA was upregulated, ICOS protein (frequency and absolute number) remained showing no statistically significant difference between LAG3⁺ and LAG3⁻ subsets post infection (Fig. 3A, I, J; Supplementary Fig. S2B). Notably, markedly increased levels of CD69 and Ki-67 (Fig. 3K, M; Supplementary Fig. S2B, C) indicated that the LAG3⁺ population was in a highly activated and actively proliferating state. After *Plasmodium* infection, LAG3⁺CD4⁺ T cells exhibited enhanced production of IL-10 and IFN-γ, while TGF-β production remained stable (Fig. 3O, Q, S; Supplementary Fig. S3A). Analysis of absolute numbers revealed that LAG3⁻ cells contributed a greater total number of IFN-γ⁺ and IL-10⁺ cells (Fig. 3P, R, T), reinforcing the distinct roles of these two subsets. To characterize the differentiation status of LAG3^+^CD4^+^ T cells, we analyzed CD44 and CD62L expression profiles [35]. Following infection, these cells exhibited a significant phenotypic shift toward an effector state. The proportion of the effector phenotype (CD44^hi^CD62L^lo^), characterized by rapid antigen responsiveness and immediate effector function, was significantly increased. Conversely, the proportion of the resting phenotype (CD44^lo^CD62L^hi^) was significantly decreased (Fig. 3U; Supplementary Fig. S3B). Therefore, *Plasmodium*-induced splenic LAG3⁺CD4⁺ T cells suggest a potential for increased functional activity during *Plasmodium* infection. Despite the higher per-cell cytokine production in LAG3⁺ cells, the LAG3⁻ population remains the primary contributor to the total cytokine pool owing to its larger numerical size.

### LEF1 binds to the *Lag3* promoter and regulates its expression

**Fig. 4:**
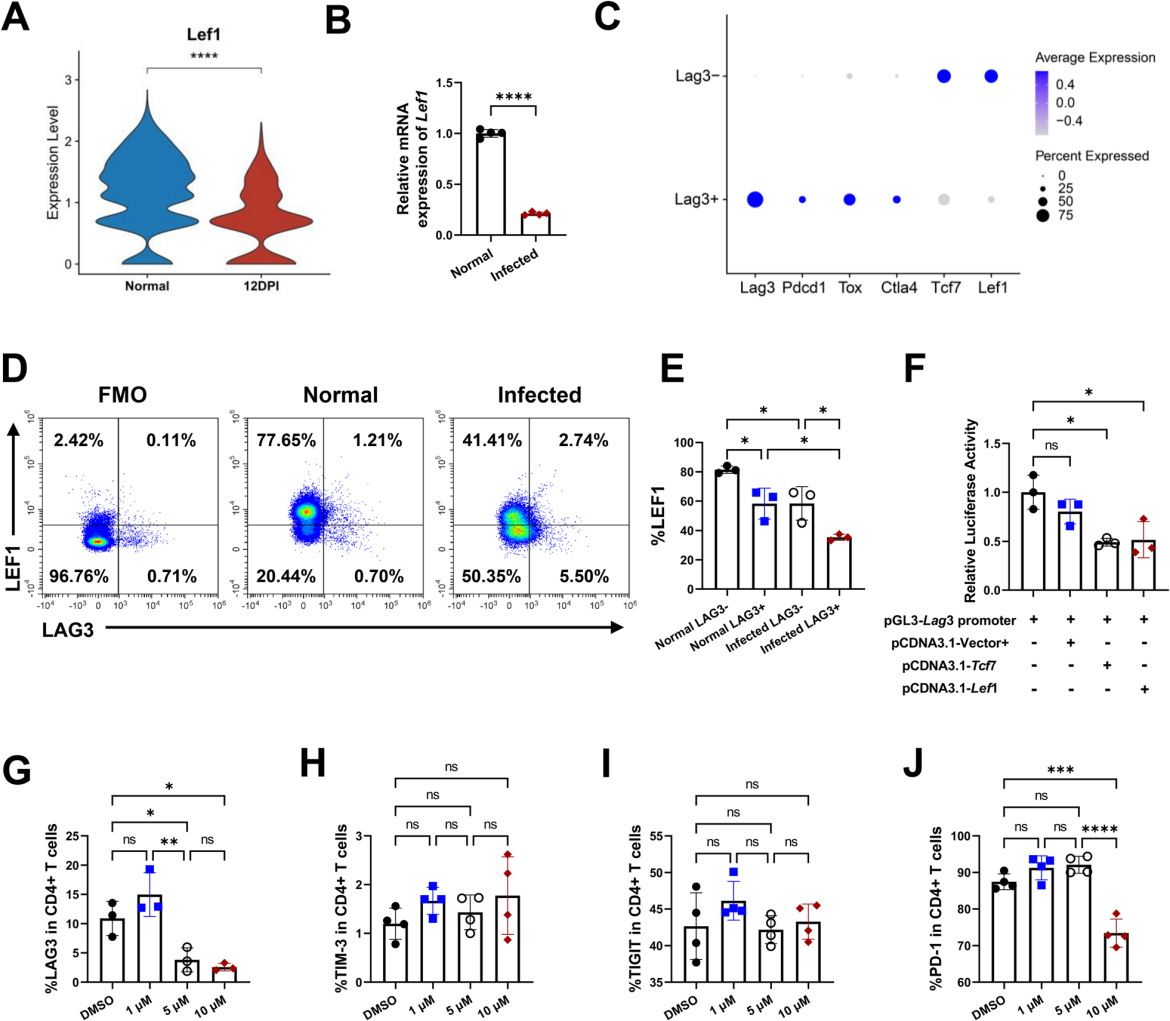
LEF1 binds to the *Lag3* promoter sequence and regulates its expression. **A** Violin plot of *Lef1* expression in CD4⁺T cells from scRNA-seq data between normal and 12 dpi groups. **B** *Lef1* mRNA level in splenic CD4⁺ T cells. **C** Dot plot of gene expression differences of *Lag3*, *Pdcd1*, *Tox*, *Ctla4*, *Tcf7*, and *Lef1* between LAG3⁻ and LAG3⁺ CD4⁺ T cells. **D**, **E** The expression of LAG3 and LEF1 in CD4⁺ T cells. **F** HEK293T cells showing *Lag3* promoter activity following co-transfection with a *Lef1* expression vector (pCDNA3.1-Lef1), a *Tcf7* expression vector (pCDNA3.1-Tcf7), or empty vector control by dual-luciferase reporter assay. **G**–**J** The expression of LAG3, TIM-3, TIGIT, and PD-1 in splenic CD4⁺ T cells following in vitro stimulation (anti-CD3/CD28) and treatment with indicated concentrations of the Wnt agonist CHIR99021. **B, E**–**J** *n* = 3–4 samples per group. Data are shown as the mean ± SD of three independent experiments. ^*^P < 0.05; ^**^P < 0.01; ^****^P < 0.0001; ns, no significant, *P* > 0.05. **A** Wilcoxon test; **B** Two-tailed unpaired *t*-test; **E**–**J** One-way ANOVA

On the basis of the activated phenotypic characteristics of LAG3⁺CD4⁺ T cells, the regulatory mechanism of LAG3 expression was further explored. DEGs analysis initially revealed a significant downregulation of the transcription factor-encoding gene *Lef1* in LAG3⁺ cells compared with LAG3⁻CD4⁺ T cells (Fig. 3A), suggesting LEF1 may act as a transcriptional repressor of *Lag3*. To validate this hypothesis, violin plots and RT–qPCR confirmed *Lef1* downregulation in splenic CD4⁺ T cells post infection (Fig. 4A, B). Furthermore, bubble plot and flow cytometry demonstrated a consistent inverse correlation between LEF1 and LAG3: LEF1 protein levels were significantly lower in LAG3⁺ cells than in LAG3⁻ cells, particularly following infection (Fig. 4C–E).

To determine whether LEF1 regulates *Lag3* transcription, we performed dual-luciferase reporter assays. Co-transfection of HEK 293 T cells with plasmids encoding *Lef1*, its co-factor *Tcf7*, and a *Lag3*-promoter reporter resulted in a significant decrease in relative luciferase activity (Fig. 4F). Although *Tcf7* mRNA levels showed an increasing trend in splenic CD4⁺ T cells post infection without statistical significance (Supplementary Fig. S4A), our data suggest that LEF1 downregulation is a significant component of *Lag3* de-repression. These findings suggest that LEF1 is capable of suppressing *Lag3* promoter activity.

To functionally validate this regulatory axis, we assessed whether activating LEF1 signaling could attenuate LAG3 upregulation. Splenic lymphocytes were stimulated with anti-CD3/CD28 antibodies and treated with CHIR99021, a GSK-3 inhibitor that stabilizes *β*-catenin to activate Wnt/LEF1 signaling [36]. Treatment with CHIR99021 resulted in a dose-dependent decrease in the frequency of LAG3⁺ cells in CD4^+^ T cells (Fig. 4G; Supplementary Fig. S4B). Notably, this effect appeared relatively specific to LAG3, as CHIR99021 did not significantly alter the expression of TIM-3 or TIGIT (Fig. 4H, I, Supplementary Fig. S4C, D), and only affected PD-1 at the highest concentration (10 μM) (Fig. 4J; Supplementary Fig. S4E). These results suggest that pharmacological activation of the Wnt/LEF1 signaling can modulate the transcriptional upregulation of LAG3.

## Discussion

The spleen, as a pivotal lymphoid organ, plays critical innate and adaptive immune responses during the *Plasmodium* erythrocytic stage to limit parasitic dissemination [37]. In our study, infection with *P. yoelii* NSM induced a peak iRBC burden at 12–16 dpi (Fig. 1A), concomitant with profound splenic alterations, including splenomegaly and elevated spleen-to-body weight ratios (Fig. 1B, C). These pathological features likely reflect not only direct tissue damage from parasite deposition but also congestion due to retention of uninfected erythrocytes, a mechanism previously implicated in malarial anemia [38]. Critically, these alterations are indicative of a massive recruitment and activation of immune cells within the splenic microenvironment.

CD4⁺ T cells orchestrate anti-malarial responses through Th1-driven phagocyte activation and Tfh-mediated antibody production [39]. Consistent with established models [40], we observed the proportional frequency of CD4⁺ T cells slightly decreased, their absolute numbers expanded significantly (Fig. 1D–F), underscoring the necessity of reporting total cell counts alongside frequencies to avoid underestimating the magnitude of the immune response. Phenotypic analysis confirmed effector differentiation, marked by the upregulation of ICOS and CD69 alongside the downregulation of CD62L (Fig. 1G–J). While ICOS induction underscores the potential role of these cells in supporting germinal center reactions [41], CD69 elevation and CD62L shedding denote early activation and lymphoid egress, respectively [42, 43]. Furthermore, cytokine profiling revealed a concomitant increase in the proportions of IFN-γ⁺ and IL-10⁺ CD4⁺ T cells (Fig. 1K–N). Such a profile may reflect a homeostatic strategy, where IFN-γ facilitates macrophage-mediated parasite clearance [44], while IL-10 potentially serves to mitigate collateral tissue damage and restrict excessive inflammation [45]. Stable TGF-β secretion suggests a homeostatic strategy, balancing Th17 induction for anti-parasite immunity with Treg modulation to prevent excessive tolerance [46].

LAG3 is a critical co-inhibitory receptor that limits excessive T cell activation, thereby maintaining the delicate equilibrium between anti-malarial immunity and host tolerance [47]. However, the functional significance of LAG3 during *Plasmodium* infection remains incompletely known. In this study, scRNA-seq and flow cytometry data suggest that LAG3 is significantly upregulated on splenic CD4⁺ T cells, peaking synchronously with parasitemia (Fig. 2). Phenotypically, these LAG3⁺ cells manifested a “highly activated and actively proliferating” profile, characterized by high expression of Ki-67, ICOS, and CD69, and co-expression of PD-1, TIM-3, and TIGIT, alongside the secretion of IFN-γ and IL-10. These characteristics align closely with phenotypes reported in mice infected with *P. yoelii* 17X and other rodent *Plasmodium* species [25, 48, 49]. This consistency across strains and models suggests that the acquisition of this phenotype is a generalizable adaptive response to *Plasmodium* infection. Crucially, this profile may represent an activated regulatory state that potentially balances the restraint of immunopathology without compromising effector function, a critical adaptation for survival in acute malaria [50]. Indeed, while LAG3⁺ cells appear more active on a “per-cell” basis, the numerically superior LAG3⁻ population likely provides the bulk of the total splenic effector response, while the LAG3⁺ subset acts as a specialized immunomodulatory rheostat. Such a functional partition provides a potential framework for understanding the balanced orchestration of parasite clearance and tissue preservation during malaria.

Mechanistically, we suggest that the LEF1–LAG3 axis functions as a potential regulator pathway in this response. DEG analysis and subsequent validation revealed a consistent inverse correlation between the transcription factor LEF1 and LAG3 expression (Fig. 4A–E). LEF1 is a key mediator of Wnt signaling, typically associated with the maintenance of T cell stemness and the naïve state [28, 51]. Our dual-luciferase assays confirmed that LEF1 directly binds to and represses the *Lag3* promoter (Fig. 4F). Although its co-factor TCF-1 (encoded by *Tcf7*) can cooperate with LEF1, our data suggest that the infection-induced downregulation of LEF1 is a key factor contributing to *Lag3* de-repression. Considering these findings, we propose a model wherein *Plasmodium* infection reduces LEF1, thereby alleviating the transcriptional repression of *Lag3* to fine-tune CD4⁺ T cell activation. Unlike chronic infection models where LEF1 deficiency drives exhaustion [52, 53], our results suggest that the LEF1–LAG3 axis participates in the functional remodeling of T cells during *Plasmodium* infection.

The potential biological implications of the LEF1–LAG3 axis warrant further investigation. Conventional checkpoint blockade (e.g., anti-LAG3 antibodies) has been associated with the risk of inducing hyperinflammation during acute infection [23, 54]—our findings suggest the possibility of a more nuanced modulation approach. Rather than a complete blockade, stabilizing LEF1, perhaps via Wnt signaling agonists like CHIR99021, could potentially maintain a functional threshold of LAG3-mediated regulation while preserving effector responses (Fig. 4G, Supplementary Fig. S4B). Notably, in our in vitro assays, this intervention appeared relatively selective for LAG3 compared with other checkpoints like TIM-3 and TIGIT. This preliminary observation distinguishes the LEF1-mediated pathway from broad, pan-checkpoint inhibitors that often disrupt immune homeostasis [55], offering a conceptual basis for future exploration of targeted immune-tuning in malaria.

Despite these insights, this study has limitations. First, while we utilized fluorescence minus one (FMO) controls and standardized gating to mitigate the impact of autofluorescence, a known challenge in malaria research, the inherent high autofluorescence of CD4⁻ splenocytes may still introduce minor variability in quadrant gating. Future studies employing additional exclusion markers or higher-resolution spectral flow cytometry would further refine these populations. Second, our murine model provides a foundational mechanism, yet the translational relevance to human *P. falciparum* or *P. vivax* infections requires validation in clinical cohorts. Third, the LEF1–LAG3 axis was validated primarily through pharmacological stabilization and luciferase assays. The use of CD4-specific *Lef1* conditional knockout mice would be necessary to definitively confirm the in vivo requirement of this axis. Finally, the potential crosstalk between LEF1 and other infection-induced transcription factors (such as Blimp-1 or Tox) remains unexplored and warrants further investigation to map the complete regulatory landscape of LAG3 in malaria.

## Conclusions

This study demonstrates that *P. yoelii* NSM infection induces profound phenotypic and functional remodeling of splenic CD4⁺ T cells, identifying a LAG3⁺ subset with both activated and regulatory signatures. Mechanistically, LEF1 acts as a transcriptional repressor of *Lag3* and its infection-induced downregulation contributes to the de-repression of LAG3 expression. The identification of the LEF1–LAG3 axis provides a conceptual framework for understanding immune homeostasis in malaria and offers a potential avenue for developing precision immunomodulatory strategies.

## Supplementary Information

Supplementary Material 1.

## Data Availability

Data supporting the main conclusions of this study are included in the manuscript.
