# Supplementary material for: The LEF1–LAG3 axis regulates CD4+ T cell function during Plasmodium yoelii NSM infection
Source: Parasit Vectors. 2026 May 26;19:264. doi: 10.1186/s13071-026-07439-5 (PMC13317295; doi:10.1186/s13071-026-07439-5)
Supplement: Supplementary file 1 — Supplementary Material 1. [file 13071_2026_7439_MOESM1_ESM.docx]

**
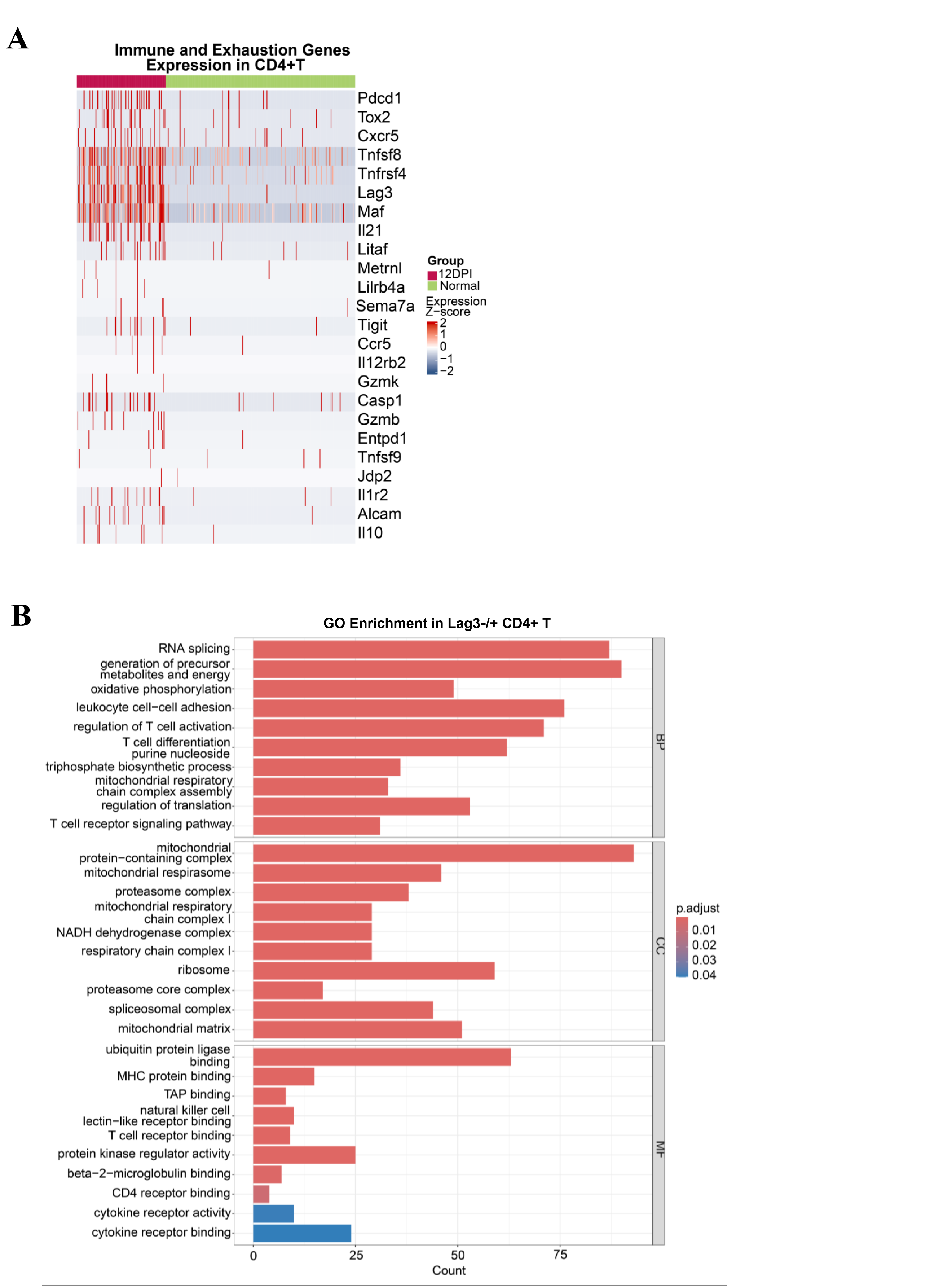
Fig. S1.** **Transcriptomic profiling of CD4⁺ T cell** **subsets.** (A) Heatmap of DEGs in splenic CD4⁺ T cells between normal and 12 dpi groups. Each column represents an individual single cell, and each row represents a gene. (B) GO pathway enrichment of DEGs between LAG3⁻ and LAG3⁺ CD4⁺ T cells.

**
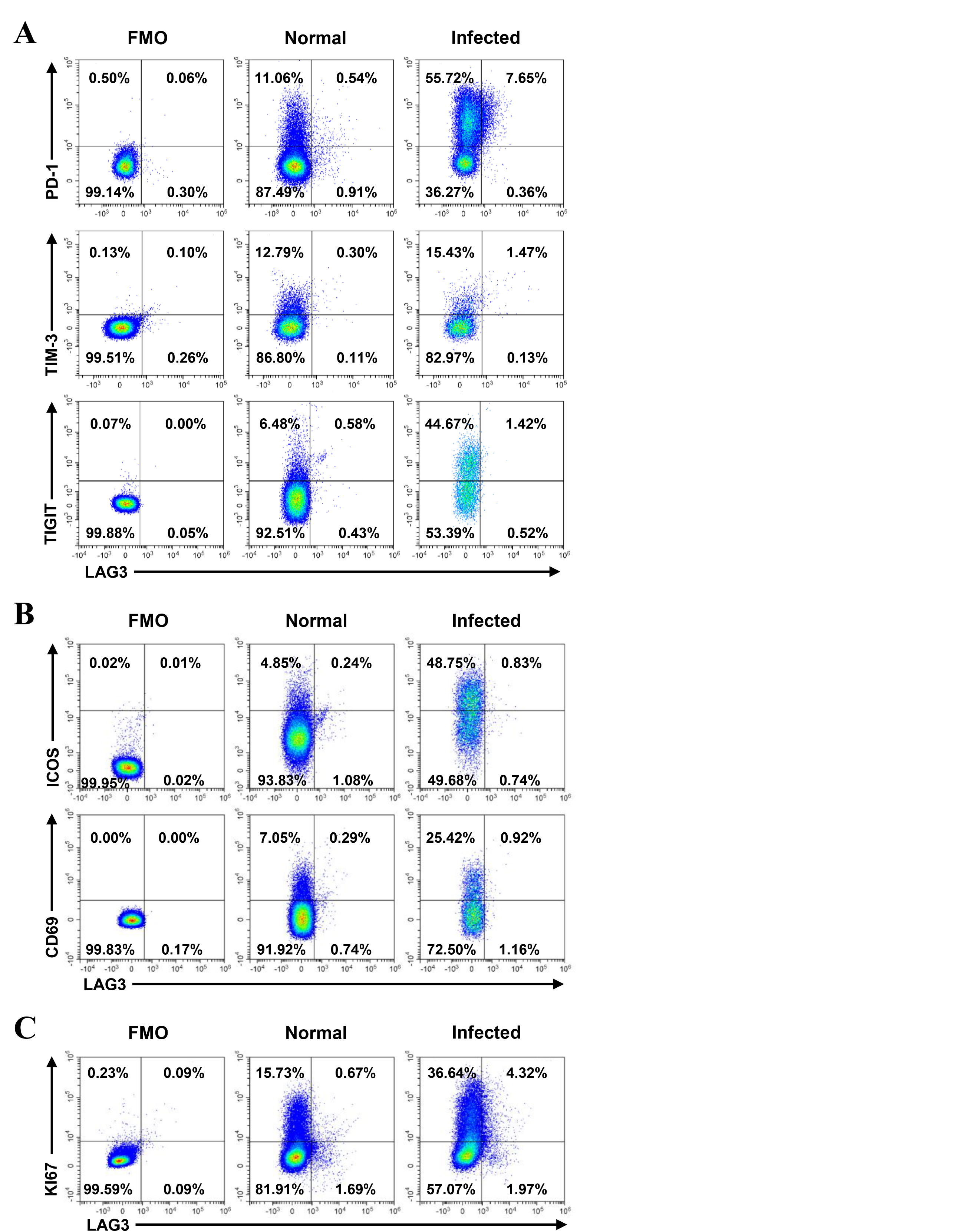
 Fig. S2. Representative flow cytometry plots of LAG3^+^ CD4⁺ T cell phenotypes.** (A) The co-expression of LAG3 with inhibitory receptors PD-1, TIM-3, and TIGIT in CD4⁺ T cells. (B) The expression of ICOS and CD69 with LAG3 in CD4⁺ T cells. (C) The percentage of Ki67 with LAG3 in CD4⁺ T cell. *Note: The apparent visual discrepancy in the frequency of the LAG3^+^ population is due to the use of different fluorophore conjugates (Brilliant Violet 660™ vs. APC). Differences in fluorophore stain index and panel-specific fluorescence spreading error affect the resolution of dim LAG3^+^ cells. Therefore, comparisons of LAG3 expression should only be made internally within the same staining panel.*

**
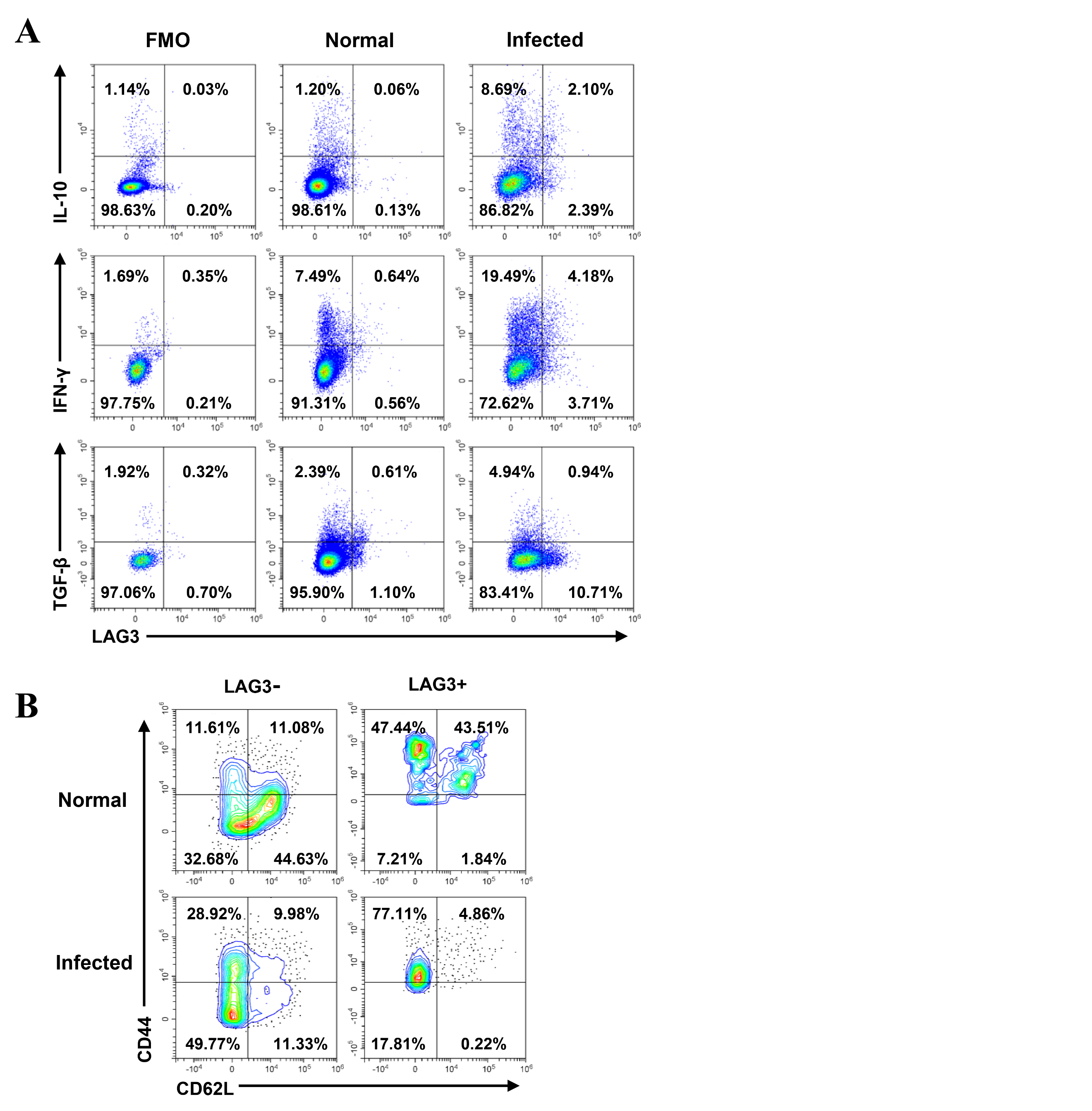
 Fig. S3. Flow cytometry gating strategy.** (A) Representative flow cytometry plots showing the expression of IL-10, IFN-γ and TGF-β between LAG3⁻ and LAG3⁺ CD4⁺ T cells. (B) Representative flow cytometry plots showing the expression of effector (CD44^hi^CD62L^lo^) and resting phenotypes (CD44^lo^CD62L^hi^) in LAG3⁻ and LAG3⁺ CD4⁺ T cells.

**
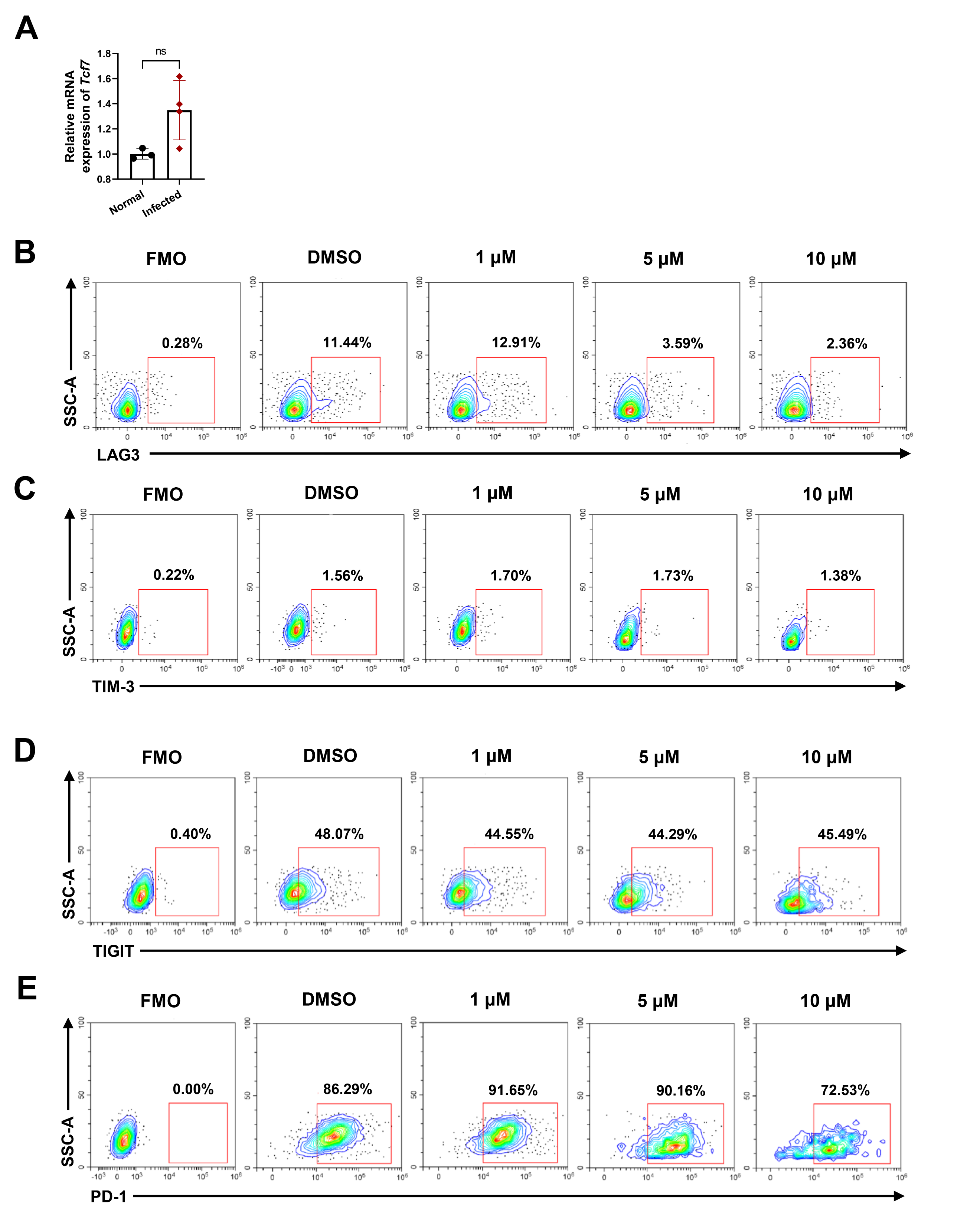
 Fig. S4. LEF1 binds to the *Lag3* promoter sequence and regulates its expression.**  (A) *Tcf7* mRNA level in splenic CD4⁺ T cells from normal and infected mice. (B-E) The expression of LAG3, TIM-3, TIGIT and PD-1 in splenic CD4⁺ T cells following *in vitro* stimulation (anti-CD3/CD28) and treatment with indicated concentrations of the Wnt agonist CHIR99021. Data are shown as the mean ± SD of three independent experiments. ns, no significant, *p* > 0.05. (A) Two-tailed unpaired t test.
